# Supplementary material for: The Parkinson’s disease drug entacapone disrupts gut microbiome homeostasis via iron sequestration
Source: Nat Microbiol. 2024 Nov 21;9(12):3165–83. doi: 10.1038/s41564-024-01853-0 (PMC11602724; doi:10.1038/s41564-024-01853-0)
Supplement: Supplementary file 1 — Supplementary Text. [file 41564_2024_1853_MOESM1_ESM.pdf]

nature microbiology

Supplementary information

https://doi.org/10.1038/s41564-024-01853-0

The Parkinson's disease drug entacapone disrupts gut microbiome homeostasis via iron sequestration

In the format provided by the authors and unedited

Supplementary Text

SRS-FISH setup optimization

To further improve the SRS-FISH sensitivity and throughput, we have upgraded the platform[1] in two major aspects. To increase sensitivity, we have switched from femtosecond SRS to picosecond SRS by spectral focusing[2,3] (Extended Data Fig.3; see Methods). Compared with femtosecond SRS, picosecond SRS allows the use of a higher laser power without damaging microbiota cells (Extended Data Fig.4a). Picosecond SRS also improves the ratio between the vibrational signal and the cross-phase modulation background when measuring Raman vibrational peaks with relatively narrow bandwidth. This enhancement is crucial for a more precise quantification of C-D bonds in metabolically active cells, that are formed through biotic incorporation of deuterium from heavy water (Extended Data Fig.4). The picosecond SRS pump power on sample is 30 mW, Stokes power on sample is 120 mW. Moreover, to increase the throughput when screening bacteria tagged by FISH, we combined point scanning SRS with widefield fluorescence imaging, which improved the screening efficiency, especially for rare cells.

Origin and heterogeneity of non-vibrational resonant signal

To understand the non-vibrational resonant signal that we observed in the ENT-Hi treated samples, we first evaluated the optical properties of pure entacapone. Entacapone is an orange coloured, non-fluorescent compound exhibiting an absorption peak at 397 nm (Extended Data Fig.6a). Under the laser illumination of the SRS microscope, there are four types of light-matter interactions that potentially exist. That includes: vibrationally-resonant SRS, non-resonant cross-phase modulation (XPM), as well as electronically-resonant transient absorption and multiphoton photothermal (PT) that could be attributed to the 397 nm absorption peak of entacapone (Extended Data Fig. 6a). As SRS and XPM are instantaneous, the signal is only generated when the pump and probe pulses are temporally overlapped (Extended Data Fig. 6b). Transient absorption is mediated by an electronic excited state with a lifetime of picosecond to nanosecond level, thus when tuning the delay time between the pump and probe pulses, the signal decays with a time constant on such a time scale[4]. The decay time of a PT signal is usually on the microsecond level[5], much longer than SRS, XPM and transient absorption. When the measurement is carried out at >1 MHz frequency, such long decay time could shift the phase of the signal to the y channel of the lock-in amplifier[6,7] (Extended Data Fig. 6b, c), while optical delay (<30ps) could not affect the signal.

By evaluating the signal from ENT-Hi cells, we observed that when changing the delay time between pump and probe pulses, the x channel output of the lock-in amplifier resembles the cross-correlation function between pump and probe pulses, which is characteristic for an instantaneous signal such as SRS and/or XPM, while an almost constant signal was observed in the y channel, which is consistent with the long decay time of a PT signal (Extended Data Fig. 6d). Taken together, these results confirm that PT is the dominating origin of the signal (Extended Data Fig. 6d).

Interestingly, we note that the PT signal, which we attributed to ENT-Hi bioaccumulation, is heterogeneous, with some cells within a particular taxon accumulating varying amounts of the drug. This can be explained by phenotypic heterogeneity due to stochastic gene expression or to different local levels of entacapone encountered by different cells, as our incubations were performed without agitation to better mimic the gut environment.

References

1. Ge, X. *et al.* SRS-FISH: A high-throughput platform linking microbiome metabolism to identity at the single-cell level. *Proc Natl Acad Sci USA* **119**, e2203519119 (2022).
2. Hellerer, T. *et al.* Monitoring of lipid storage in Caenorhabditis elegans using coherent anti-Stokes Raman scattering (CARS) microscopy. *Proc Natl Acad Sci USA* **104**, 14658–14663 (2007).
3. Fu, P. *et al.* Super-resolution imaging of non-fluorescent molecules by photothermal relaxation localization microscopy. *Nat Photon* **17**, 330–337 (2023).
4. Zhu, Y. & Cheng, J.-X. Transient absorption microscopy: Technological innovations and applications in materials science and life science. *J Chem Phys* **152**, 020901 (2020).
5. Bai, Y., Yin, J. & Cheng, J.-X. Bond-selective imaging by optically sensing the mid-infrared photothermal effect. *Sci Adv* **7**, eabg1559 (2021).
6. Zhang, D., Slipchenko, M. N., Leaird, D. E., Weiner, A. M. & Cheng, J.-X. Spectrally modulated stimulated Raman scattering imaging with an angle-to-wavelength pulse shaper. *Opt Express* **21**, 13864–13874 (2013).
7. Samolis, P. D. & Sander, M. Y. Phase-sensitive lock-in detection for high-contrast mid-infrared photothermal imaging with sub-diffraction limited resolution. *Opt Express* **27**, 2643–2655 (2019).
