# Supplementary material for: The Parkinson’s disease drug entacapone disrupts gut microbiome homeostasis via iron sequestration
Source: Nat Microbiol. 2024 Nov 21;9(12):3165–83. doi: 10.1038/s41564-024-01853-0 (PMC11602724; doi:10.1038/s41564-024-01853-0)
Supplement: Supplementary file 2 — Reporting Summary [file 41564_2024_1853_MOESM2_ESM.pdf]

nature portfolio

Corresponding author(s): Dr. Fatima C. Pereira
Prof. Michael Wagner
Prof. Ji-Xin Cheng

Last updated by author(s): Sep 30, 2024

Reporting Summary

Nature Portfolio wishes to improve the reproducibility of the work that we publish. This form provides structure for consistency and transparency in reporting. For further information on Nature Portfolio policies, see our Editorial Policies and the Editorial Policy Checklist.

Statistics

For all statistical analyses, confirm that the following items are present in the figure legend, table legend, main text, or Methods section.

n/a	Confirmed	
☐	☒	The exact sample size (*n*) for each experimental group/condition, given as a discrete number and unit of measurement
☐	☒	A statement on whether measurements were taken from distinct samples or whether the same sample was measured repeatedly
☐	☒	The statistical test(s) used AND whether they are one- or two-sided<br>*Only common tests should be described solely by name; describe more complex techniques in the Methods section.*
☐	☒	A description of all covariates tested
☐	☒	A description of any assumptions or corrections, such as tests of normality and adjustment for multiple comparisons
☐	☒	A full description of the statistical parameters including central tendency (e.g. means) or other basic estimates (e.g. regression coefficient) AND variation (e.g. standard deviation) or associated estimates of uncertainty (e.g. confidence intervals)
☐	☒	For null hypothesis testing, the test statistic (e.g. *F*, *t*, *r*) with confidence intervals, effect sizes, degrees of freedom and *P* value noted<br>*Give P values as exact values whenever suitable.*
☒	☐	For Bayesian analysis, information on the choice of priors and Markov chain Monte Carlo settings
☒	☐	For hierarchical and complex designs, identification of the appropriate level for tests and full reporting of outcomes
☐	☒	Estimates of effect sizes (e.g. Cohen's *d*, Pearson's *r*), indicating how they were calculated

Our web collection on statistics for biologists contains articles on many of the points above.

Software and code

Policy information about availability of computer code

Data collection	BD FACSChorus™ v3.0; Minknow v. 21.10.8, Oxford Nanopore Technologies; Leica Application Suite X (LAS X 5.1.0).
Data analysis	ARB v. 7.0; R v4.0.2; R packages: DADA2 v1.16.0, vegan v2.5-.6, phyloseq v1.30.0, DESeq2 v1.26.0; Demultiplex FASTA/FASTQ v1.2.1; cutadapt v. 3.1; Minimap2 v. 2.17; flye v. 2.9-b1768; Racon v. 1.4.3; Medaka v. 1.4.4; SAMtools v. 1.12; MetaBAT2 v. 2.15; QUAST v. 5.0.2; CheckM v. 1.1.1; GTDBtk v. 1.5.1; Barrnap v. 0.9; trnascan v. 2.0.6; INFERNAL v. 1.1.3; AMRFinderPlus v.3.10.21; IQTREE v 1.6.12; rrnDB (version 5.7); ImageJ software v 1.53t; FeGenie (v.1.2); Cell Profiler v4.2.6; Matlab R2023a, Fiji 1.54g with OpenMIMS plug in v3.0.5.<br>In addition, CellProfiler pipelines and Matlab codes have been deposited in GitHub (https://github.com/buchenglab/srs-fish-drugs)

For manuscripts utilizing custom algorithms or software that are central to the research but not yet described in published literature, software must be made available to editors and reviewers. We strongly encourage code deposition in a community repository (e.g. GitHub). See the Nature Portfolio guidelines for submitting code & software for further information.

Data

Policy information about availability of data

All manuscripts must include a data availability statement. This statement should provide the following information, where applicable:

- Accession codes, unique identifiers, or web links for publicly available datasets
- A description of any restrictions on data availability
- For clinical datasets or third party data, please ensure that the statement adheres to our policy

16S rRNA gene sequencing data, metagenomics data and retrieved MAGs have been deposited in the National Center for Biotechnology Information (NCBI) Sequence Read Archive under BioProject number PRJNA1033532.
Databases used were: Genome Taxonomy Database (GTDB) v.0.1.3 (https://gtdb.ecogenomic.org/); RDP taxonomy 18, release 11.5 (https://doi.org/10.5281/zenodo.4310151); Short Read Archive (https://www.ncbi.nlm.nih.gov/sra); SILVA taxonomy (release 138) using the DADA2 classifier (https://zenodo.org/records/4587955); SILVA 119 SSU NR99 database (https://www.arb-silva.de/download/arb-files/).

Research involving human participants, their data, or biological material

Policy information about studies with human participants or human data. See also policy information about sex, gender (identity/presentation), and sexual orientation and race, ethnicity and racism.

Reporting on sex and gender	This study included participants of both sexes: 3 males and 6 females. In most of the analyses performed, samples from both sexes were combined (i.e. pooled), and therefore we could not perform analyses with sex as a factor. In experiments where samples were analysed separately (long-read sequencing of faecal samples), there was insufficient statistical power to perform analyses with sex as a factor.
Reporting on race, ethnicity, or other socially relevant groupings	N.a.
Population characteristics	Study participants (between 22 and 39 years old, average age 32.4 years old) had not received antibiotics in the prior 3 months and had no history of digestive disease.
Recruitment	Study participants were researchers of the University of Vienna and University of Southampton. Participants were recruited via email. Participants worked in the same building and were chosen based on their availability to provide a fresh faecal sample. All study participants provided written informed consent. Participants did not receive any compensation from participating in this study. Participants were all young adults, which can be considered a sampling bias.
Ethics oversight	University of Vienna Ethics Committee (reference #00161). University of Southampton Ethics and Research Governance Office (reference #78743).

Note that full information on the approval of the study protocol must also be provided in the manuscript.

Field-specific reporting

Please select the one below that is the best fit for your research. If you are not sure, read the appropriate sections before making your selection.

☒ Life sciences ☐ Behavioural & social sciences ☐ Ecological, evolutionary & environmental sciences

For a reference copy of the document with all sections, see nature.com/documents/nr-reporting-summary-flat.pdf

Life sciences study design

All studies must disclose on these points even when the disclosure is negative.

Sample size	For microcosms experiments, faecal samples form 6 individuals were pooled and used per incubation, and each condition was run in triplicates. All triplicates were sequenced and analysed. Our rationale for mixing samples from 6 healthy donors was to assess the effect of drugs on a microbial community with greater microbial diversity than that of a single individual. For feasibility reasons (availability of freshly collected samples on the same day), this number was restricted to six donors. No statistical methods were used to pre-determine sample sizes, but our sample sizes are similar to those reported in previous publications. For SRS analyses, the number of cells analyzed was chosen based on feasibility.
Data exclusions	No other data was excluded from analyses, except for:<br>-amplicon sequencing variants (ASVs, from 16S rRNA gene amplicon sequencing) assigned to Cyanobacteria or chloroplast were removed based on pre-established criteria: these ASVs were detected in negative controls but were not present in any of the faecal samples, and were therefore removed from analyses;<br>-stimulated Raman scattering (SRS) analyses: during image acquisition and processing, outlier cells displaying abnormal intensity (>mean±2 standard deviations) were rejected from the single cell masks. This intensity threshold was set after test and validation with independent samples, as food residues can be distinguished based on an irregular shape compared to cells, and by a stronger signal due to other absorption processes, that will gradually decrease due to the degradation of the absorption component.

Replication	Microcosm experiments with drugs were replicated (two times in total) and the results were successfully reproduced. Entacapone and iron rescue experiments using pure culture experiments were successfully reproduced one time. All other experiments were replicated at least one time. NanoSIMS analysis were only performed once based on feasibility, and was deemed successful because the iron levels in entacapone-exposed cells were very highly significant compared to control cells. For SRS measurements, we reproducibly detected analogous differences between treatments when sample analyses were repeated.
Randomization	For microcosms experiments, faecal samples were thoroughly mixed prior to allocation to microcosms, and allocations to microcosms were performed randomly. For flow cytometry, cell counts and SRS/photothermal analyses, both sample preparation and sample measurements were randomized. Preparation of samples for sequencing was randomized. For NanoSIMS, we first analysed the entacapone treated samples followed by the untreated controls to confirm that the observed Fe distribution patterns were not affected by measurement artifacts.
Blinding	Blinding during the microscosm and pure culture experiments was not possible, as different samples had to receive a different treatment/ drug. Sample preparation for sequencing was carried out by an independent investigator in a blinded manner. Initial steps of sequencing data processing were performed blindly. Bioinformatic analyses of sequencing data was performed with automated software, but were not performed blindly, as we needed to know which samples to compare. For all other experiments or assays, the investigators were not formally blinded because of the high demand for certain sample/group samples at certain time points, which required appropriate selection of some of the samples.

Reporting for specific materials, systems and methods

We require information from authors about some types of materials, experimental systems and methods used in many studies. Here, indicate whether each material, system or method listed is relevant to your study. If you are not sure if a list item applies to your research, read the appropriate section before selecting a response.

Materials & experimental systems

n/a	Involved in the study
☒	☐ Antibodies
☒	☐ Eukaryotic cell lines
☒	☐ Palaeontology and archaeology
☒	☐ Animals and other organisms
☒	☐ Clinical data
☒	☐ Dual use research of concern
☒	☐ Plants

Methods

n/a	Involved in the study
☒	☐ ChIP-seq
☐	☒ Flow cytometry
☒	☐ MRI-based neuroimaging

Plants

Seed stocks	N.a.
Novel plant genotypes	N.a.
Authentication	N.a.

Flow Cytometry

Plots

Confirm that:

☒ The axis labels state the marker and fluorochrome used (e.g. CD4-FITC).

☒ The axis scales are clearly visible. Include numbers along axes only for bottom left plot of group (a 'group' is an analysis of identical markers).

☒ All plots are contour plots with outliers or pseudocolor plots.

☒ A numerical value for number of cells or percentage (with statistics) is provided.

Methodology

Sample preparation	Faecal samples from six donors (two males, four females) were transferred into an anaerobic tent (Coy Laboratory Products, USA) within 30 min after sampling, and all sample manipulation and incubations were performed under anaerobic conditions (5% H2, 10% CO2, 85% N2). Each sample was suspended in M9 mineral medium supplemented with 0.5 mg.mL-1 D-glucose

(Merck), 0.5% v/v of vitamin solution (DSMZ Medium 461) and trace minerals, herein referred to as sM9. Samples were suspended in sM9 to yield a 0.05 g.mL-1 faecal slurry. At this point one aliquot of each sample was collected, pelleted, and stored at -80°C for metagenomic analysis. The homogenate was left to settle for 10 minutes, and the supernatant (devoid of any large faecal particles) was transferred into a new flask, where supernatants from the six different donors were combined. This combined sample was further diluted 1:10 in sM9 medium (as described above) or in supplemented Brain Heart Infusion (BHI) medium containing either 0% or 55% D2O (99.9% atom % (at%) D; Merck) for final 0% (control) or 50% D2O in incubation medium. Supplemented BHI medium consisted of 37 g.L-1 of brain heart infusion broth (Oxoid), 5 g.L-1 yeast extract (Oxoid), 1 g.L-1 L-cysteine (Merck) and 1 g.L-1 NaHCO3 (Carl Roth GmbH, Germany). Incubation tubes were supplemented with dimethylsulfoxide (DMSO, from Merck), entacapone (Prestwick Chemicals) or loxapine succinate (Prestwick Chemicals) pre-dissolved in DMSO. The final concentration was 2% w/v of DMSO in all vials (except for the H2O control, where water was added instead of DMSO). A subset of vials was supplemented with 20 µM or 1965 µM entacapone (ENT-Low and ENT-Hi, respectively) and another subset was supplemented with 20 µM or 100 µM loxapine succinate (LOX-Low and LOX-Hi, respectively). At time 0, and after an incubation time of 6 or 24 hours at 37°C under anaerobic conditions, two sample aliquots from each incubation and controls were collected by centrifugation. One aliquot was washed with 1× PBS and then fixed in 3% paraformaldehyde solution for 2 h at 4°C. Samples were finally washed two times with 1 ml of PBS and stored in PBS:Ethanol (50% v/v) at -20°C until further use. The second pelleted aliquot was stored at -20°C until further processing. A third aliquot was collected into sealed anaerobic vials containing 40% glycerol (Carl Roth GmbH, Germany) in PBS for a final cell 50% v/v cell suspension in 20% glycerol and stored at -80°C until further use. In addition, amendment of ENT-Hi to three individual faecal samples (two females and one male) and sample processing were carried as described above, except that samples were not mixed prior to incubation.
Samples preserved in glycerol were diluted 200 to 800 times in 1x PBS. To remove any additional debris from the faecal incubations, samples were transferred into a flow cytometry tube by passing the sample through a snap cap containing a 35 µm pore size nylon mesh. Next, 500 µL of the microbial cell suspension was stained with the nucleic acid dye SYTO™ 9 (Thermo Fisher Scientific, 0.5 µM in DMSO) for 15 min in the dark. The flow cytometry analysis of the microbial cells present in the suspension was performed using a BD FACSMelody™ (BD Biosciences), equipped with a BD FACSChorus™ software v 3.0 (BD Biosciences).

Instrument

BD FACSMelody™ (BD Biosciences)

Software

Collection: BD FACSChorus™ software v 3.0 (BD Biosciences).
Analyses and visualisation: Microsoft Excel v 16.87 was used for data sorting and R v 4.0.2 was used for data visualization.

Cell population abundance

No cells were sorted.

Gating strategy

Briefly, background signals from the instrument and the buffer solution (PBS) were identified using the operational parameters forward scatter (FSC) and side scatter (SSC). Microbial cells were then displayed using the same settings in a scatter plot using the forward scatter (FSC) and side scatter (SSC) and pre-gated based on the presence of SYTO™ 9 signals. Singlets discrimination was performed. Absolute counting beads (CountBrightTM, ThermoFisher Scientific) added to each sample were used to determine the number of cells per mL of culture by following the manufacturer's instructions. Fluorescence signals were detected using the blue (488 nm – staining with SYTO™ 9 and CountBright™ beads) and yellow-green (561 nm - CountBrightTM beads only) optical lasers. The gated fluorescence signal events were evaluated on the forward–sideways density plot, to exclude remaining background events and to obtain an accurate microbial cell count. Instrument and gating settings were identical for all samples (fixed staining-gating strategy).

☒ Tick this box to confirm that a figure exemplifying the gating strategy is provided in the Supplementary Information.
